# Recurrence of Asymptomatic COVID-19 after Recovery among Healthcare Workers

**DOI:** 10.1155/2022/8787867

**Published:** 2022-03-19

**Authors:** Cucunawangsih Cucunawangsih, Ratna Sari Wijaya, Nata Pratama Hardjo Lugito, Ivet Suriapranata

**Affiliations:** Faculty of Medicine, Pelita Harapan University, Tangerang, Indonesia

## Abstract

We describe five healthcare workers (HCWs) with a recurrence of asymptomatic SARS-CoV-2 infection at Siloam Teaching Hospital, Indonesia. All cases involved nurses, with an average age of 27 years. The RT-PCR assay confirmed the first and second infection episodes. All cases showed negative RT-PCR results in the period between two infection episodes. The median interval time between two infection episodes was 123 days, ranging from 92 to 158 days. The clinical outcomes for all cases were favourable, with no mortality observed among study cases. Further studies will be required to understand the true nature of this phenomenon.

## 1. Introduction

Coronavirus disease 2019 (COVID-19), caused by severe acute respiratory syndrome-coronavirus 2 (SARS-CoV-2), was first detected in China in December 2019, and it has spread rapidly worldwide. The first COVID-19 case in Indonesia was confirmed on March 2, 2020, and there were approximately 3,908,247 cases, including 121,141 deaths and 343,203 active cases as of August 18, 2021 [1]. Indonesia has recorded the highest number of confirmed and active COVID-19 cases among Southeast Asian countries [2].

The first report of SARS-CoV-2 reinfection cases confirmed by genome sequencing was described in Hong Kong [3]. Since then, reports of genetically characterized reinfections have emerged [4, 5], raising major public health concerns about the degree and duration of protective immunity against SARS-CoV-2 infection. Considering it is not always practicable to confirm the reinfection case by viral genomic testing, we have used the term “recurrence” to describe the possible reinfection case. While most individuals with COVID-19 recurrence showed symptoms in one or both episodes, we report the recurrence of positive SARS-CoV-2 RNA in asymptomatic COVID-19 healthcare workers (HCWs) detected during routine surveillance. Given that asymptomatic HCWs may shed a considerable viral load and play a significant role in nosocomial and community transmission, this study highlights the importance of regular testing among HCWs in the healthcare system.

## 2. Case Presentation

This retrospective study was conducted in the Siloam Teaching Hospital, Indonesia, from October 1, 2020, until March 31, 2021. A monthly combined screening, including reverse-transcriptase polymerase chain reaction (RT-PCR) on nasopharyngeal swabs and serology test for total antibodies against the SARS-CoV-2 nucleocapsid (anti-N) (Elecsys anti-SARS-CoV-2, Roche Diagnostics, Switzerland), was performed in the period October 2020 to March 2021, on all HCWs in the Siloam Teaching Hospital, Indonesia. Epidemiological, clinical, laboratory, and outcome data of HCWs with recurrence of COVID-19 were obtained from the hospital's database. The following criteria were used for selecting HCWs with asymptomatic COVID-19 recurrence during this period of study: (1) no prior exposure to SARS-CoV-2, as indicated by negative for total anti-N antibodies at the initiation time of study; (2) an initial SARS-CoV-2 RNA detection by reverse-transcriptase polymerase chain reaction (RT-PCR); (3) followed by clinical recovery with at least one negative SARS-CoV-2 RT-PCR result; (4) followed by the second time of SARS-CoV-2 RNA RT-PCR positive result ≥90 days after the initial positive test; and (5) asymptomatic in both episodes. The recurrence asymptomatic COVID-19 cases identified through the retrospective study were screened and asked for consent.

The cases presented had two episodes of SARS-CoV-2 infection, confirmed by positive SARS-CoV-2 RNA detection in nasopharyngeal swab specimens using reverse-transcriptase polymerase chain reaction (RT-PCR) between October 2020 and March 2021, at Siloam Teaching Hospital, Indonesia. Demographic, clinical, and laboratory data for each case, including the time interval between the first and second episode of infection, are summarized in Table 1.

In this first episode of infection, all cases were asymptomatic and detected during regular RT-PCR testing among HCWs conducted between October and December 2020. A test that detected a single target gene N or ORF1ab (BioA SARS-CoV-2 RT-PCR kit, BioAcumen, Singapore) with a cycle threshold (Ct) < 40 was considered as positive. The median Ct value of the N gene and ORF1ab gene from the first infection was 32.4 (range 29.6–37.7) and 34.5 (range 31.2–37.8), respectively. The median duration of the detectable virus was 18 days (range 16–30 days) before testing negative for SARS-CoV-2 by RT-PCR. The mean age of cases was 27 years (±3.8 years). All the cases were female and worked as nurses. No comorbidities were identified among the cases in this study. The clinical outcome from the first episode of infection was favourable for all cases, as no hospitalization was required, and no mortality was observed among cases.

The interval time of RT-PCR between the first and second infection ranged from 92 to 158 days, with a median interval time of 123 days. The second infection episodes were detected during regular RT-PCR testing conducted between January and March 2021. The median Ct value of the N gene and ORF1ab gene in the second infection was 33.2 (range 32.1–34.4) and 33.9 (range 32.9–35.1), respectively. All cases were asymptomatic until the negative RT-PCR result occurred 9 to 21 days (median 21 days) after the second positive RT-PCR result. Similar to the first episode of infection, all cases had a favourable clinical outcome in the second infection.

**Figure 1:**
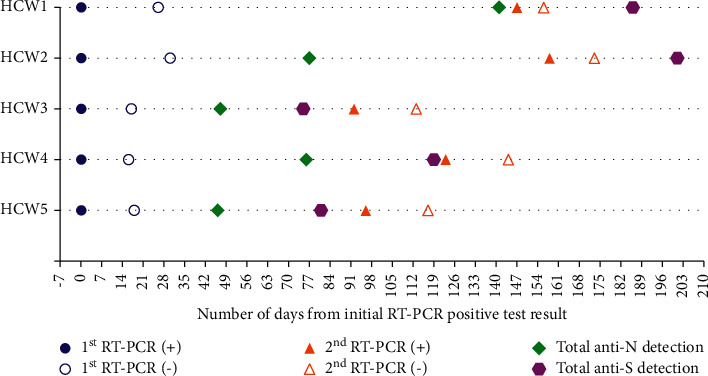
Testing timeline of healthcare workers (HCWs) with recurrence episodes of asymptomatic COVID-19 infection, Siloam Teaching Hospital, Indonesia, October 2020 to March 2021. HCW, healthcare workers; RT-PCR, reverse-transcriptase polymerase chain reaction; N nucleocapsid; S spike; +, positive; -, negative.

All cases had serology testing performed by using Elecsys anti-SARS-CoV-2 and Elecsys anti-SARS-CoV-2 S assays (Roche Diagnostics, Switzerland) that detect total antibodies against recombinant nucleocapsid (N) and recombinant spike (S) SARS-CoV-2 protein receptor-binding domain (RBD) SARS-CoV-2, respectively. After the first episode, all cases had positive total antibodies against the N SARS-CoV-2 protein that tested from 46 to 141 days (median 76 days) from the first positive RT-PCR. Total antibodies against S protein were detected in HCW1 and HCW2 after the second episode of infection, whereas in HCW3, HCW4, and HCW5, antibodies were observed before the second episode of infection (Figure 1).

## 3. Discussion

This study reported five cases of recurrence asymptomatic COVID-19, confirmed by positive SARS-CoV-2 RNA testing results conducted ≥90 days apart, with a negative molecular test result in the interim period. The incidence of recurrent SARS-CoV-2 positivity among individuals who have recovered from COVID-19 has been reported ranging from 7.6% to 48.9% [6–8]. A recent systematic review and meta-analysis including 41 studies on COVID-19 recurrence cases worldwide have estimated that 15% of patients were repositive for SARS-CoV-2 RNA after discharge [9]. Moreover, the high detection of repositive asymptomatic cases has been reported in South Korea study, accounted for 56.6% (158 out of 284) of cases [6].

All current cases worked as nurses, which has been considered a risk factor that is significantly associated with recurrence of COVID-19 disease due to prolonged exposure to suspected or confirmed COVID-19 patients [10, 11]. The younger age that was observed in all cases has also increased the risk for recurrence positivity [8, 11]. The clinical outcome of all COVID-19 recurrence cases was favourable, as all the cases did not require hospitalization, and no one succumbed during the first and second episodes of infection. The absence of underlying medical conditions observed and the younger age among participants in the current study potentially contributed to the nonsevere recurrence cases, similar to previous findings [3, 12]. In contrast, the severe disease during the second episode of infection has also been observed in prior studies [10, 13]. The presence of risk factors for severe illness such as older age and comorbidities among study participants in these prior studies likely explains the discrepancy of recurrence of COVID-19 cases' clinical outcome [10, 13].

Similar to previous reports [14, 15], high Ct values (>30) were observed in asymptomatic cases, indicating a low viral load. The finding that all five HCWs with COVID-19 recurrence were asymptomatic and had low viral loads is noteworthy and may possibly indicate the inadequate immune response induced by infection episodes to prevent further recurrent infection [16]. In addition, the absence of symptoms suggests the high possibility of undetected recurrence of SARS-CoV-2 infection and, subsequently, amplifying the spread of COVID-19 disease in healthcare and community settings [15].

The antibody against the N SARS-CoV-2 protein (anti-N) was observed in all cases after the first infection episode, confirming the presence of SARS-CoV-2 infection. In the case of SARS-CoV-2 antibodies, the most important neutralizing action is the antibody against the RBD region of S1 (anti-S), given its function in binding to targeted host cells [17]. The recurrent COVID-19 was observed in three HCWs (HCW3, HCW4, and HCW5) that had shown different anti-S antibody concentrations before the second RT-PCR positive results, suggesting other factors such as cellular immunity and virus variant may contribute to the recurrence of COVID-19.

Although the viral genomic testing was not performed in this study to further investigate the SARS-CoV-2 genetic profile, the recurrence events among HCWs were indicated by the following: (1) the negative RT-PCR result between two episodes of SARS-CoV-2 infection, ruling out the possibility of persistent viral shedding; (2) the prolonged interval time between the first and second RT-PCR (≥90 days), indicating the higher suspicion for the recurrence of COVID-19; and (3) the presence of antibodies against SARS-CoV-2 protein, confirming the infection episode.

Considering the challenges in identifying asymptomatic COVID-19 cases [18], and the current routine surveillance may not completely detect the asymptomatic infection cases among HCWs, the number of recurrent asymptomatic COVID-19 cases during this study period is possibly underreported. The presence of asymptomatic recurrent COVID-19 disease among HCW in the current study highlighted the importance of multiple rounds of RT-PCR testing in routine surveillance to identify the recurrence of asymptomatic cases and prevent further nosocomial and community transmission. In addition, while the level and duration of postinfection immunity in asymptomatic individuals with recurrent infection remain unclear, mitigation to protect this population from the SARS-CoV-2 exposure should be implemented. COVID-19 vaccination and preventive measures are particularly important to optimally protecting these high-risk individuals.

## Figures and Tables

**Table 1:** Clinical and laboratory characteristics among five healthcare workers (HCWs) with a recurrence of asymptomatic COVID-19 disease, Siloam Teaching Hospital, Indonesia.

No.	Sex	Age	Profession	Comorbidities	Vaccine	1^st^ infection				Interval between 1^st^ and 2^nd^ positive rt-PCR	2^nd^ infection				Serology	
						Clinical feature	Outcome^a^	Ct-value	RT-PCR negative day^b^	↔	Clinical feature	Outcome^a^	Ct-value	RT-PCR negative day^b^	Anti-N^c^	Anti-S^c^
1	F	24	Nurse	No	No	Asymp	Favourable	N: 32.4 ORF1ab: neg	D26	147	Asymp	Favourable	N: 32.1 ORF1ab: 32.9	D9	R (D141)	>250 (D186)
2	F	28	Nurse	No	No	Asymp	Favourable	N: 31.2 ORF1ab: neg	D30	158	Asymp	Favourable	N: 32.2 ORF1ab: 35.1	D15	R (D77)	>250 (D201)
3	F	33	Nurse	No	No	Asymp	Favourable	N: 37.7 ORF1ab: 37.8	D17	92	Asymp	Favourable	N: 33.2 ORF1ab: neg	D21	R (D47)	>250 (D75)
4	F	25	Nurse	No	No	Asymp	Favourable	N: 29.6 ORF1ab: 31.2	D16	123	Asymp	Favourable	N: 33.2 ORF1ab: neg	D21	R (D76)	162.2 (D119)
5	F	24	Nurse	No	No	Asymp	Favourable	N: 32.6 ORF1ab: neg	D18	96	Asymp	Favourable	N: 34.4 ORF1ab: neg	D21	R (D46)	51.6 (D81)

Ct, cycle threshold; Asymp, asymptomatic; neg, negative; R, reactive; NR, nonreactive. ^a^Clinical outcome was evaluated by the requirement of hospitalization and survival of COVID-19 patients. ^b^Considering the day of RT-PCR positive as day 0. ^c^Considering the day of RT-PCR positive on first infection as day 0.

## Data Availability

The data used to support the findings of this study are included within the article.
